# *Kdm3b* haploinsufficiency impairs the consolidation of cerebellum-dependent motor memory in mice

**DOI:** 10.1186/s13041-021-00815-5

**Published:** 2021-07-03

**Authors:** Yong Gyu Kim, Myeong Seong Bak, Ahbin Kim, Yujin Kim, Yun-Cheol Chae, Ye Lee Kim, Yang-Sook Chun, Joon-Yong An, Sang-Beom Seo, Sang Jeong Kim, Yong-Seok Lee

**Affiliations:** 1 Department of Physiology, Seoul National University College of Medicine, Seoul, 03080 Korea; 2 Department of Biomedical Sciences, Seoul National University College of Medicine, Seoul, 03080 Korea; 3 Department of Biosystems and Biomedical Sciences, College of Health Sciences, Korea University, Seoul, 02841 Korea; 4 Department of Integrated Biomedical and Life Sciences, College of Health Sciences, Korea University, Seoul, Korea; 5 Department of Life Science, College of Natural Science, Chung-Ang University, Seoul, 06974 Korea; 6 Ischemic/Hypoxic Disease Institute, Seoul National University College of Medicine, Seoul, 03080 Korea; 7 Neuroscience Research Institute, Seoul National University College of Medicine, Seoul, 03080 Korea

**Keywords:** *Kdm3b*, Optokinetic response (OKR), Histone modification, Cerebellum

## Abstract

Histone modifications are a key mechanism underlying the epigenetic regulation of gene expression, which is critically involved in the consolidation of multiple forms of memory. However, the roles of histone modifications in cerebellum-dependent motor learning and memory are not well understood. To test whether changes in histone methylation are involved in cerebellar learning, we used heterozygous *Kdm3b* knockout (*Kdm3b*^+/−^) mice, which show reduced lysine 9 on histone 3 (H3K9) demethylase activity. H3K9 di-methylation is significantly increased selectively in the granule cell layer of the cerebellum of *Kdm3b*^+/−^ mice. In the cerebellum-dependent optokinetic response (OKR) learning, *Kdm3b*^+/−^ mice show deficits in memory consolidation, whereas they are normal in basal oculomotor performance and OKR acquisition. In addition, RNA-seq analyses revealed that the expression levels of several plasticity-related genes were altered in the mutant cerebellum. Our study suggests that active regulation of histone methylation is critical for the consolidation of cerebellar motor memory.

**Supplementary Information:**

The online version contains supplementary material available at 10.1186/s13041-021-00815-5.

## Introduction

One of the hallmarks of memory consolidation is that it requires changes in gene expression [1, 2]. Since DNA is tightly packaged with histone proteins to form chromatin in the nucleus, chromatin structure should be remodeled to induce gene activation or repression by epigenetic mechanisms, such as modifications of histone proteins and DNA [3]. It has been shown that epigenetic regulation, such as histone modification and DNA methylation, is a key mechanism for memory consolidation from invertebrates to mammals [4, 5].

Post-translational modifications of histone proteins, such as acetylation and methylation, determine whether the gene is in a transcriptionally permissive or repressive state [6, 7]. Methylated histones are generally considered codes for gene repression, but their effects on transcription are very complex [8]. For example, tri-methylation of histone 3 lysine 9 (H3K9me3) represents a repressive code, whereas H3K4me3 is considered an activating code [9]. Histone methylation is dynamically regulated by many histone methyltransferases and demethylases [10]. Lysine demethylase 3b (*KDM3B*) encodes a demethylase that targets mono-and di-methylated lysine 9 on histone 3 (H3K9me1/2) [11]**.** KDM3B has been shown to regulate many cellular processes, including gene expression in leukemia, spermatogenesis, and autophagy [11–13]. Recent studies have shown that *KDM3B* mutations are associated with cognitive deficits, including intellectual disability and schizophrenia [14, 15]. However, the functional role of KDM3B in the nervous system remains unclear.

The cerebellum plays a central role in adaptive motor control. For newly formed motor memory to be expressed in an appropriate circumstance, consolidation of motor memory by the cerebellum is essential [16]. Similar to other types of learning, the consolidation of cerebellum-dependent motor memory also requires de novo transcription and translation [17]. However, the epigenetic mechanisms underlying cerebellar learning and memory have rarely been investigated. In this study, we investigated how changes in histone methylation affect cerebellum-dependent motor behavior and gene expression in the cerebellum. Using *Kdm3b* heterozygous knockout (*Kdm3b*^+/−^) mice, we found deficits in retention of cerebellum-dependent motor memory in the mutant mice, demonstrating that regulation of histone methylation in the cerebellum is critical for cerebellum-dependent memory consolidation.

## Materials and methods

### Animals

Adult (> 7-week-old) male *Kdm3b*^+/−^ mice and their wild-type (WT) littermates were used. *Kdm3b*-targetted ES cell line (YTC406) was obtained from International Gene Trap Consortium (https://igtc.org/cgi-bin/annotation.py?cellline=YTC406). Genomic sequencing was performed to identify the targeting vector (pGT0lxf) insertion site in the ES cell line. The heterozygous knockout mice were generated by injecting the ES cells and backcrossed to C57Bl/6N background (Macrogen, Inc). Genotypes of the mice were identified by PCR using the following primers: common forward 5′-GGC ACC AGA CCC TGG GAG CTA G-3′, WT reverse 5′-CAC CCA CGA CCT GGC TTA CAC C-3′, KO reverse 5′-CAC CCA CGA CCT GGC TTA CAC C-3′. The mutant mice were crossed to C57Bl/6J 3–4 times before the experiments. Animals were housed with food and water available ad libitum under a 12-h light/dark cycle. All animal experiments were performed in accordance with the protocols approved by the Animal Care and Use Committee of Seoul National University.

### Tissue sample preparation

To prepare the granule cell-enriched (GCE) and Purkinje-cell-enriched (PCE) tissue samples, sagittal slices of the cerebellar vermis (250 μm thick) were obtained using a vibratome (VT1200, Leica). Vermal slices were manually dissected into GCE and PCE samples under a stereomicroscope, as previously described [18].

### Histone purification

In order to extract purified histone protein from tissues, tissues were homogenized with TEB buffer (0.5 × Triton X-100, 2 mM PMSF, 1 × Protease inhibitor cocktail) at 4 °C for 30 min. Homogenized tissues were centrifuged at 7,000 rpm for 2 min at 4 °C. After removing the supernatant, the pellet was washed with additional TEB buffer and centrifuged under the same conditions. After removing the supernatant, 0.5 M HCl was added to the pellet. The tube was rotated overnight at 4 °C. After centrifugation at 13,500 rpm for 10 min at 4 °C, the supernatant (~ 90% of the total volume) was transferred to a fresh tube. Subsequently, 1/3 volume of 100% TCA was added to the tube, which was inverted and placed on ice for 2 h. Occasionally, the tube was vortexed. The tube was centrifuged at 13,500 rpm for 10 min at 4 °C. The supernatant was removed and acetone was added. The tube was centrifuged at 13,500 rpm for 5 min at 4 °C. Acetone was air-dried and distilled water was added. The tube was agitated overnight at 4 °C. The tube was centrifuged at 13,500 rpm for 5 min at 4 °C. The supernatant containing the histone extract was then saved.

### Western blot analyses

Western blot analysis was performed as described previously [19]. Briefly, tissues were homogenized and sonicated with RIPA buffer (25 mM Tris–HCl [pH 7.6], 150 mM NaCl, 1% NP-40, 1% sodium deoxycholate, 0.1% SDS) with a phosphatase inhibitor cocktail and a protease inhibitor cocktail. Proteins were quantified using bicinchoninic acid (BCA) analysis according to the manufacturer’s instructions (Pierce). Either 3 μg of histone or 20 μg of total tissue extract were used for Western blot analyses. The membranes were imaged with a digital imager (Amersham Imager 600, GE Life Science). Protein quantification was performed using the AI600 software (GE Healthcare), and each band was normalized to α-tubulin or β-actin in the same gel. The following primary antibodies and dilution ratios were used: anti-KDM3B antibody (2621S, 1:500; Cell Signaling Technology), anti-H3 antibody (Millipore, 05-499, 1:2,000), anti-H3K9me2 antibody (Millipore, 07-441, 1:1,000), anti-α-tubulin antibody (Santa Cruz, TU-02, 1:2,000), and anti-β-actin antibody (Santa Cruz, AC-15, 1:2,000).

### Behavioral test

The whole procedure, including surgical and behavioral processes, was performed according to a previously described method with minor modifications [18, 20]. Briefly, a headpost was mounted on the head of the mouse using nuts, screws, and dental cement. Mice were administered for at least 24 h after surgery. Optokinetic and vestibular stimuli were generated by a patterned screen and a custom-made turntable with a restrainer attached. Right eye movements were monitored using an infrared video-oculography system. The entire system was controlled by a custom-written LabVIEW (National Instruments, USA) code. To assess basal oculomotor function in mice, the optokinetic response (OKR) and vestibulo-ocular reflex in dark and light (dVOR and lVOR) were measured. The OKR was evoked by sinusoidal oscillation of the screen with a rotation amplitude of 5° and four rotational frequencies (0.1, 0.25, 0.5, and 1.0 Hz). The VOR was induced by sinusoidal oscillation of the turn-table under the same stimulation conditions for the OKR. Twelve cycles were performed for each measurement.

To induce oculomotor learning in mice, mice were subjected to a 50 min-long sinusoidal oscillation of the screen with an amplitude of 5° and frequency of 0.5. During the training session, the OKR was measured every 10 min. After the training session, mice were subjected to a consolidation session for 24 h in complete darkness. The final OKR was measured immediately after completion of the consolidation session. Data analysis for oculomotor behavior was performed as described in our prior study [21]. The gain was defined as the ratio of eye velocity to stimulus velocity. The phase was defined as the time difference between the two velocity curves.

### RNA sequencing analysis

Bulk RNA samples were collected from the GCE of the cerebellar vermis. We obtained two WT and two *Kdm3b*^+/−^ mice samples for RNA-sequencing analysis [Gene Expression Omnibus Series GSE173255 (GSM5264380–GSM5264383)]. After trimming the low-quality bases, the sequence reads were aligned using the STAR algorithm [22]. We then quantified gene expression using StringTie [23], resulting in 16,447 genes expressed in the cerebellum tissues. For differentially expressed (DEX) gene analysis, we summarized a gene-level expression from transcript-level expression using the tximport R package [24] and performed the DEX analysis using the DESeq2 R package. After removing genes without expression, we normalized the gene expression counts by the total library size. We defined DEX genes with a log_2_ fold change greater than 1.2, and with an adjusted p-value under 0.05, compared to wild type mice.

### Gene ontology (GO) analysis

To investigate the related pathways of DEX genes, we performed GO analysis using gprofiler2 [25]. GO analysis utilizes functional annotations from the GO biological process (GO:BP), cellular component (GO:CC), and molecular function (GO:MF) databases. After multiple comparisons, only significant results were collected (adjusted p < 0.05). These results were used to identify the related genes and pass on to the network construction and analysis.

### Network construction and visualization

To represent a functional relationship of the trans-synaptic signaling pathway (GO:0099177), which we found to have significant enrichment for DEX genes, we constructed a gene network using the 28 DEX genes involved in the pathway. We used Cytoscape 3.8.0 software (https://cytoscape.org/) and GeneMANIA plugin [26] to build a gene network where the nodes are the DEX genes and their first-degree interactors, and the edges are defined by physical interaction and co-expression similarity.

### Real-time quantitative PCR (RT-qPCR)

Total RNA of vermal GCE tissue or flocculus was purified sample using the RNeasy Mini Kit (QIAGEN) according to the manufacturer’s instructions. Genomic DNA was eliminated by DNase I (QIAGEN). Isolated total RNA was quantified by Nanodrop (ThermoFisher). The same amount of total RNA per sample was reverse transcribed into cDNA using SuperScript III First-Strand (Invitrogen). RT-qPCR was conducted using the CFX Connect system (Bio-Rad) with primers adopted from the previous studies *Fam107a* [27]; *Homer3* [28]; *Fgf1* [29]; *Car2* [30]; *Cplx2* [20]; *Ntf3* [31]. *Kdm3b* mRNA expression was analyzed using the following primers: 5′-CAC ATC ATC GCC TCA GTG GTA-3′ and 5′-CCC ATC GCC ATC TCC TTC AC-3′. Mean Ct values were used relative quantification of mRNA expression between two genotype or behavioral groups using the ∆∆Ct method, with the ratio of the target gene expressed relative to the mean of *Gapdh*.

### Statistics

An independent t-test and two-way repeated-measures ANOVA post-hoc Tukey test were used to evaluate the statistical significance between independent and dependent samples, respectively. All statistical computations were performed using R v3.6. software (http://www.r-project.org). The significance level was set at P < 0.05. Error bar denotes SEM.

## Results

### Increased H3K9me2 in the cerebellum of *Kdm3b*^+/−^ mice

**Fig. 1:**
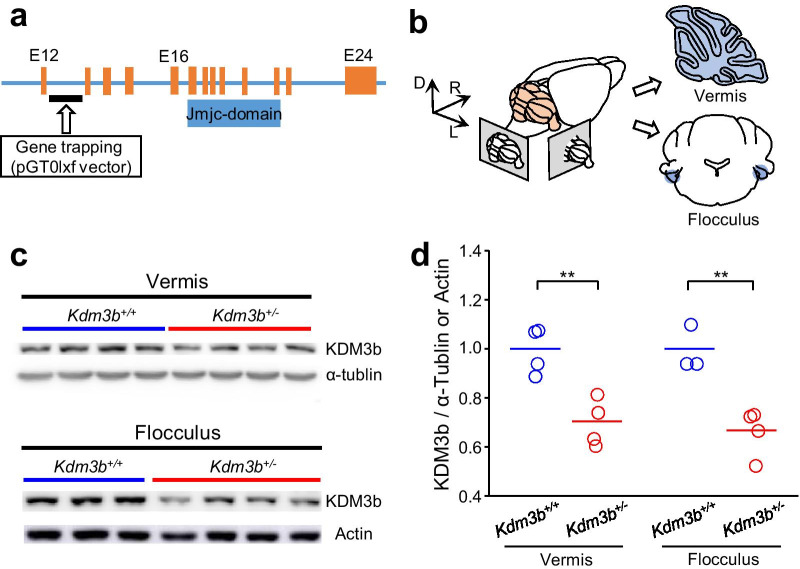
KDM3b expression is decreased in the cerebellum of *Kdm3b*^+/−^ mice. **a** Conserved domains in *Kdm3b* gene and targeting strategy by using pGT0lxf gene trapping system in introns12-13. **b** Preparation of two cerebellar regions (light blue), the verims and flocculus, for Quantification of KDM3b expression. **c** Quantification of KDM3b protein expression in the cerebellar vermis (top) and flocculus (bottom) of *Kdm3b*^+/+^ and *Kdm*3b^+/−^ mice using immunoblotting. **d** Comparison of KDM3b expression in two cerebellar regions between two genotypes. An independent t-test was performed to test statistical significance between two groups. **p < 0.01

To investigate whether the regulation of histone methylation is critically involved in cerebellum-dependent motor learning and memory, we used *Kdm3b* heterozygous knockout mice. The mutant mice were generated by inserting the pGT0lxf gene trapping vector between exons 12 and 13 of the *Kdm3b* gene (Fig. 1a). Haploinsufficiency of *Kdm3b* does not affect gross morphology of the brain (Additional file 1: Fig. S1). We obtained the cerebellar vermis and flocculus from *Kdm3b*^+/+^ and *Kdm3b*^+/−^ mice and quantified Kdm3b protein expression levels (Fig. 1b and c). We confirmed that the Kdm3b protein expression level was significantly reduced in the two cerebellar regions of the mutants compared to those in wild-type (WT) littermates (Fig. 1d; Vermis: df = 6, p < 0.01; Flocculus: df = 5, p < 0.01; independent t-test).

**Fig. 2:**
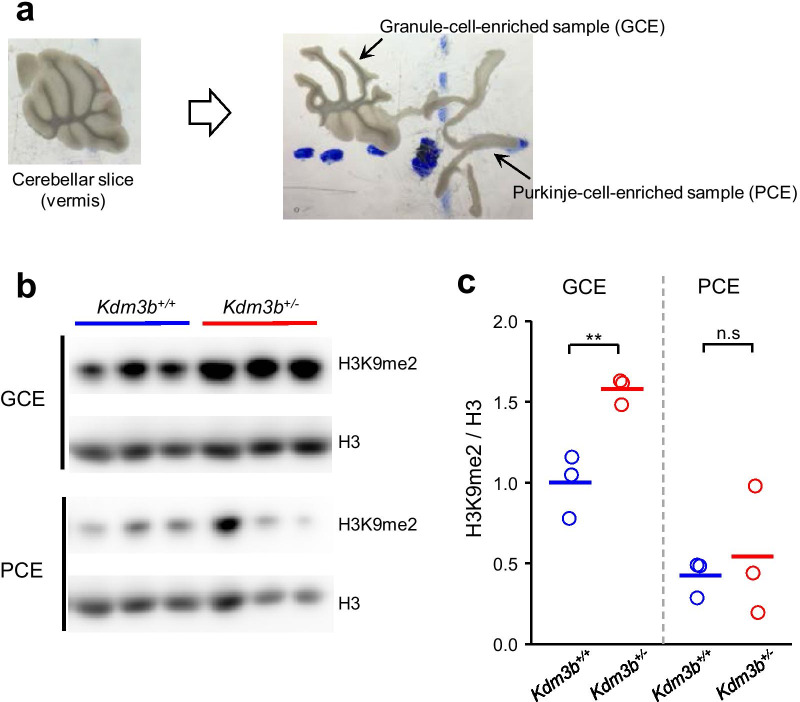
Increased H3K9me2 in the cerebellar granule cell layer of *Kdm3b*^+/−^ mice. **a** Microdissection of cerebellar vermis to separate granule-cell-enriched (GCE) and Purkinje-cell-enriched (PCE) samples. **b**, **c** Layer-specific quantification of H3K9me2 level in GCE and PCE samples from *Kdm3b*^+/+^ and *Kdm*3b^+/−^ mice. N = 3 mice per group. An independent t-test was performed to test statistical significance between two groups. **p < 0.01. n.s indicates p > 0.05

Since Kdm3b has been reported as a demethylase targeting H3K9me1 and H3K9me2 [11], we examined whether H3K9 methylation levels are altered in the cerebellum of *Kdm3b*^+/−^ mice. When we assessed H3K9me2 level in the flocculus, a crucial region for cerebellum-dependent oculomotor memory, there is no significant difference in H3K9me2 levels between genotypes (Additional file 1: Fig. S2). Given that the expression pattern of H3K9me2 in the cerebellum remains largely unknown, we attempted a more selective sampling method for quantification of H3K9me2 in the cerebellum using micro-dissection technique which allows us to detect subtle changes in molecular expression [32]. When we micro-dissected the granule-cell-enriched (GCE) and Purkinje-cell-enriched (PCE) samples in the cerebellar vermis (Fig. 2a), we found that H3K9me2 levels were significantly increased in GCE samples from *Kdm3b*^+/−^ mice compared to those from WT mice (Fig. 2b and c; df = 4, p < 0.01; independent t-test). On the other hand, there was no significant difference in H3K9me2 levels between genotypes in PCE samples (df = 4, p > 0.05; independent t-test), showing that Kdm3b regulates H3K9 methylation in the cerebellum in a layer-specific manner.

### No changes in basal oculomotor performance in ***Kdm3b***^+/−^ mice

**Fig. 3:**
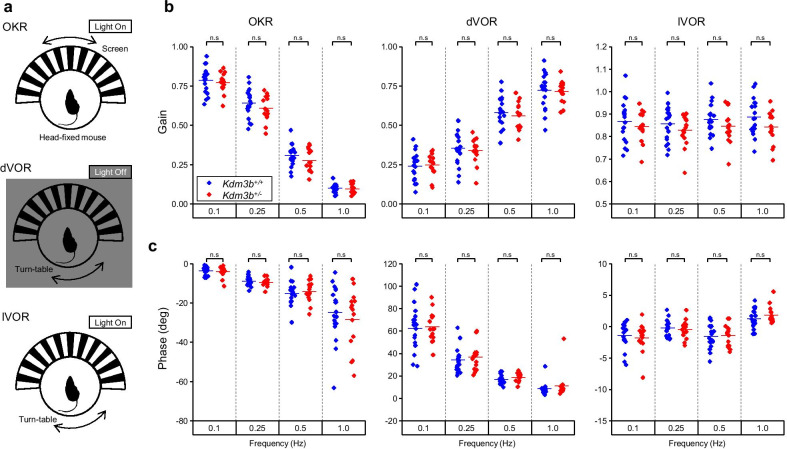
No change in basal oculomotor performance in *Kdm3b*^+/−^ mice. **a** Three classes of basal oculomotor function. Optokinetic response (OKR, top), dark (dVOR, middle) and light vestibulo-ocular reflex (lVOR, bottom). **b** Comparison of gains of OKR, dVOR, and lVOR at different stimulating frequencies between *Kdm3b*^+/+^ (n = 19 mice) and *Kdm3b*^+/−^ (n = 15 mice) groups. **c** Comparison of phases of OKR, dVOR, and lVOR at different stimulating frequencies between two groups. An independent t-test was performed to test statistical significance between two groups. n.s indicates p > 0.05

To test the effects of heterozygous knockout of *Kdm3b* on cerebellum-dependent behavior, we subjected *Kdm3b*^+/−^ mice to oculomotor adaptation, which is a simple paradigm of cerebellum-dependent motor learning [20, 33]. First, we tested the basal oculomotor performance in *Kdm3b*^+/−^ mice in comparison with their WT littermates using two classes of oculomotor reflexes, OKR and VOR. The OKR is a reflexive oculomotor response evoked by the motion of the visual field. The VOR is a compensatory oculomotor response driven by the motion of the head in a direction opposite to the head movement. In our experiment, head-fixed mice were exposed to sinusoidal oscillation of the screen or turn-table to induce OKR or VOR, respectively (Fig. 3a). *Kdm3b*^+/−^ mice showed comparable gain (strength) and phase (timing) of the OKR, and VOR both in the dark (dVOR) and light (lVOR) (Fig. 3b and c) (*Kdm3b*^+/+^, n = 19 mice; *Kdm3b*^+/−^, n = 15 mice; p > 0.05 for all comparison pairs, independent t-test), suggesting that haploinsufficiency of *Kdm3b* does not affect the basal oculomotor performance in mice.

### Cerebellum-dependent memory consolidation deficits in ***Kdm3b***^+/−^ mice

**Fig. 4:**
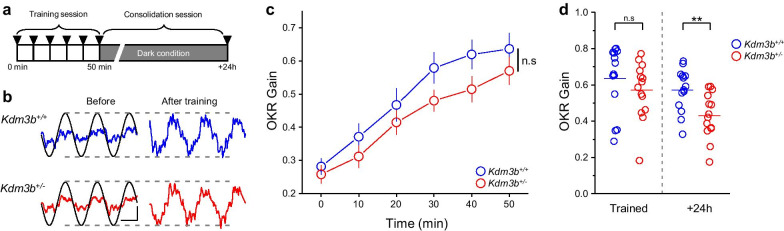
Cerebellum-dependent motor memory consolidation deficits in *Kdm3b*^+/−^ mice. **a** Experimental scheme. Eye movement was measured every 10 min in the training session, and after the consolidation session (upside-down triangles). **b** Representative optokinetic response (OKR) of *Kdm3b*^+/+^ (blue) and *Kdm3b*^+/−^ (red) mice evoked by screen oscillation (black) before and after training. **c** Changes in OKR gains of *Kdm3b*^+/+^ (n = 14 mice) and *Kdm3b*^+/−^ (n = 14 mice) groups in the training session. **d** Comparison of OKR gains in *Kdm3b*^+/+^ and *Kdm3b*^+/−^ mice before and after the consolidation sessions. A two-way repeated-measures ANOVA post-hoc Tukey test was used to test statistical significance between two groups. ** and n.s indicate p < 0.01 and p > 0.05, respectively.

Next, we investigated the ability of *Kdm3b*^+/−^ mice for cerebellum-dependent learning and consolidation. Long-term exposure of mice to the sinusoidal oscillation of an optokinetic screen induces a gradual increase in OKR gain, which is dependent on the cerebellum [34]. Here, we trained the mice under continuous oscillation of the screen for 50 min with an amplitude of 5° and frequency of 0.5 Hz (Fig. 4a). The OKR was measured before and every 10 min during the training session. To assess the level of memory consolidation, OKR gain was measured in mice after 24 h of consolidation. In the training session, *Kdm3b*^+/−^ mice exhibited slightly lower OKR gain than wild-type littermates, but this difference was not statistically significant (Fig. 3b and c; *Kdm3b*^+/+^, n = 14 mice; *Kdm3b*^+/−^, n = 14 mice; p > 0.05, two-way repeated-measures ANOVA). After training, whereas the level of OKR memory in WT littermates was well maintained up to 24 h, the level of OKR memory in *Kdm3b*^+/−^ mice was significantly reduced (Fig. 3d; Trained p > 0.05; + 24 h p < 0.01; two-way repeated-measures ANOVA with post-hoc Tukey test). Together, our results show that haploinsufficiency of *Kdm3b* leads to a significant deficit in the consolidation of cerebellar memory without affecting memory formation.

In addition to the deficit in the consolidation of oculomotor memory in *Kdm3b*^+/−^ mice, we examined whether *Kdm3b* expression is regulated by OKR learning in wildtype mice. To this end, C57Bl/6 J mice were subjected to the same behavioral procedures as before (Fig. 4a) and then sacrificed to extract total RNA from the flocculus. The RT-qPCR with reverse transcription result showed that the *Kdm3b* mRNA level was significantly decreased in trained group compared with control group (Additional file 1: Fig. S3) (df = 4, p < 0.01; independent t-test). Our results suggest that *Kdm3b* and its activity-dependent gene regulation is critically involved in the consolidation of cerebellar memory.

### Altered gene expression in the cerebellum of ***Kdm3b***^+/−^ mice

**Fig. 5:**
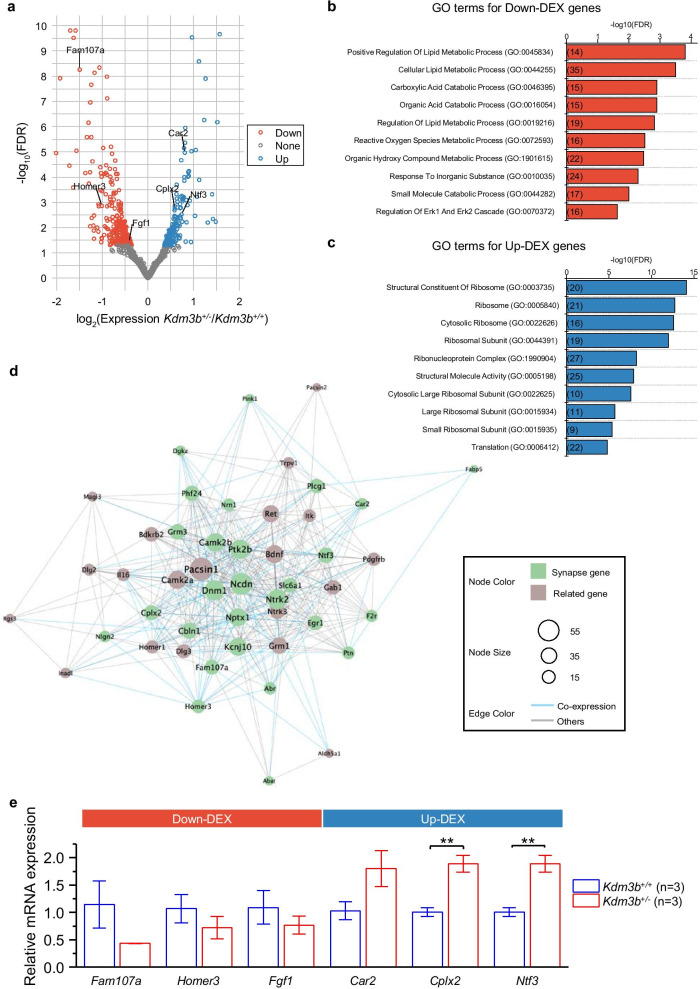
RNA-seq analysis in the cerebellar granule cell layer of *Kdm3b*^+/−^ mice. **a** Volcano plot of the differentially expressed (DEX) genes in *Kdm3b*^+/−^ mice compared to WT mice. Y axis is the false discovery rate (FDR) with − log10 used and X axis is the expression values of *Kdm3b*^+/−^ mice divided by WT mice with log2 used. Two mice per group were used for the analyses. **b** Gene ontology (GO) analysis of the down-DEX genes compared to wild type mice. Three GO domain, GO biological process (GO:BP), cellular component (GO:CC), and molecular function (GO:MF) were used. Results with term size equal to or bigger than 30 and less than 1000 were used. Y axis is the name of the terms of the GO results and X axis is the FDR with − log10 used. **c** GO analysis of the up-DEX genes compared to wild type mice. Results were filtered same as in (b). In **b** and **c**, the number in parentheses indicates the number of genes belonging to a given GO term. **d** Gene network analysis using 28 genes, which intersected with the GO term ‘regulation of trans-synaptic signaling’ (GO:0099177; p-value = 1.4E−02). The intersected genes are colored green. **e** RT-qPCR validation of selected DEX genes. RT-qPCR analysis targeted the six selected genes (*Fam107a*, *Homer3*, *Fgf1*, *Car2*, *Cplx2*, and *Ntf3*) was performed using GCE samples obtained from *Kdm3b*^+/+^ and *Kdm3b*^+/−^ mice (n = 3 per group). An independent t-test was performed to test statistical significance between two groups. **p < 0.01

To evaluate the transcriptional consequences underlying *Kdm3b* knockout, we examined differentially expressed (DEX) genes in *Kdm*3b^+/−^ mice by performing RNA sequencing. A total of 509 DEX genes were found in *Kdm*3b^+/−^ mice compared to WT mice, including 194 upregulated and 315 downregulated genes (Fig. 5a, Additional file 2: Table S1). To identify the biological functions and related pathways of DEX genes, we performed a gene ontology (GO) analysis, which revealed that DEX genes are involved in numerous biological pathways (Additional file 3: Table S2), including the regulation of trans-synaptic signaling pathway (GO:0099177; p-value = 1.4E−02). We further investigated the GO pathways by splitting DEX genes into down-DEX (n = 315) and up-DEX genes (n = 194) and found that down-DEX genes were mostly related to lipid metabolic processes. In particular, 14 down-DEX genes (*Dgkz, Akt2, Epha8, Fgf1, Fgfr3, Cyr61, Prkcd, Ptk2b, Srebf1, Slc27a1, Irs2, Angptl4, Pnpla2, Ccdc3*) are involved in the positive regulation of lipid metabolic process pathway (GO:0045834, p-value = 1.6E−04) (Fig. 5b, Additional file 3: Table S2). In contrast, up-DEX genes are mostly involved in biological pathways related to translation and biosynthetic processes. Of note, 22 genes, such as *Rpl31, Fau, Eif2ak2, Purb, and Rpl21*, were shown in the translation pathway (GO:0006412, p-value = 1.7E−05) (Fig. 5c). Among the DEX genes, 28 genes (e.g., *Fam107a, Fabp5, Ptk2b, Homer3, F2r, Egr1, Car2, Ntf3, Kcnj10, Plcg1, and Slc6a1*) were related to the trans-synaptic signaling pathway (GO:0099177; p-value = 1.4E−02) (Additional file 3: Table S2). We further assessed whether the changes in gene expression in the cerebellar GCE layer in *Kdm*3b^+/−^ mice are related to synaptic activity. We focused on the 28 genes of the trans-synaptic signaling pathway from the GO results and analyzed their relationships with other genes using gene network analysis (Fig. 5d). As a result, 20 genes were newly identified and assumed to be involved in the trans-synaptic signaling pathway. The hub gene, which is connected to the largest number of nodes, was *Pacsin1*, followed by *Ncdn, Dnm1*, and *Ntrk2*.

Finally, to validate the RNA-seq results, we performed RT-qPCR analysis using GCE samples obtained from independent groups of *Kdm3b*^+/+^ and *Kdm3b*^+/−^. Among the 28 DEX genes belonging to the GO term ‘regulation of trans-synaptic signaling’, we selected five genes: *Fam107a*, *Homer3*, *Car2*, *Cplx2*, and *Ntf3*. Three of them (*Ntf3* [35], *Cplx2* [36, 37], *Homer3* [38]) were suggested as regulators of learning and memory and *Fam107a* was known to be expressed in the cerebellar granule cell [39]. In addition to these five genes, we also attempted to validate the downregulation of *Fgf1* gene which has been suggested as a crucial gene in regulating the persistence of fear memory in the amygdala [29]. In RT-qPCR analysis, we again found significant increases in *Cplx2* and *Ntf3* mRNA levels in GCE samples of *Kdm3b*^+/−^ group compared with those of *Kdm3b*^+/+^ group (Fig. 5e; df = 5, p < 0.01; independent t-test). For the other genes, the difference in mRNA levels between the two genotypes did not reach statistical significance (p > 0.05), but the same tendency was confirmed when compared with the RNA-seq results, supporting the validity of our RNA-seq results.

## Discussion

Here, we report that haploinsufficiency of *Kdm3b* results in deficits in the consolidation of OKR memory. Moreover, we found that *Kdm3b* deletion affects the transcription of several plasticity-related genes in the cerebellum, which may also contribute to memory deficits in mutant mice.

Unlike short-term memory, which is dependent on the covalent modifications of pre-existing molecules, long-term memory requires changes in transcription and translation [1, 2, 20]. The requirement for the synthesis of new mRNA and protein for memory consolidation has been demonstrated in multiple forms of learning in diverse species including *Aplysia*, *Drosophila*, and mammals. It has been shown that infusing actinomycin D or anisomycin into the mouse cerebellar flocculus before OKR training blocks the increase in OKR gain after 4 h, but not after 1 h, suggesting that transcription and translation are also required for the consolidation of cerebellum-dependent motor learning [17]. In addition, injecting anisomycin into the cerebellar nuclei impairs the retention of conditioned response in the eye blink conditioning task, which is another well-known cerebellar learning paradigm [40]. Recently, Kim et al. have shown that cerebellum-dependent learning triggers changes in protein expression in the cerebellum in a learning paradigm-dependent manner [20]. In addition, the late phase of cerebellar long-term depression, which is considered a cellular mechanism for certain forms of cerebellar learning, was also shown to be dependent on protein synthesis [41]. Despite accumulating evidence for the critical roles of epigenetic regulation on gene expression and memory [4], surprisingly, whether epigenetic mechanisms are involved in cerebellar motor learning has not yet been investigated.

Active changes in histone methylation in response to learning or other external stimuli have been observed in multiple brain regions [42–46]. In particular, H3K9me2 was increased in the hippocampus, entorhinal cortex, and amygdala 1 h after mice were trained in the fear conditioning paradigm [42–44]. The expression of the histone lysine methyltransferase G9a increased after fear conditioning, which might be responsible for the increased H3K9me2 [44]. Consistently, inhibiting G9a impaired fear memory, while pharmacological inhibition of the histone lysine demethylase LSD1 (KDM1A) enhanced fear memory [44]. This result is interesting since we found that haploinsufficiency of *Kdm3b* impairs, rather than enhances, motor memory. A recent study showed that another H3K9 demethylase, plant homeodomain finger protein 2 (PHF2), acts as a positive regulator of hippocampal memory without affecting amygdala-dependent cued fear memory [47], suggesting that each histone demethylase may have a distinct role depending on brain regions and behavioral tasks. It would be interesting to examine the role of other histone-modifying enzymes, including LSD1, in cerebellar learning.

We identified several genes that were differentially expressed in the cerebellar granule cell-enriched layer, which may contribute to the OKR consolidation deficit in *Kdm3b*^+/−^ mice. Among DEX genes, *Cbln1* (log2 fold change = 4.0E−01; p-value = 3.6E−02) was previously reported to be secreted in the cerebellar granule cells and plays an essential role in controlling synaptic structure and plasticity [48]. In addition, *Ntf3* (log2 fold change = 7.4E−01; p-value = 3.0E−03), which encodes neurotrophin-3, is mostly secreted by mature granular cells and contributes largely to cerebellar development, such as Purkinje cell maturation [49]. Although we showed that *Kdm3b* haploinsufficiency did not affect the gross morphology of the cerebellum (Additional file 1: Fig. S1), whether *Kdm3b* deletion have any subtle effects on the brain development remains to be examined. One of the other DEX genes, *Ptn* (log2 fold change = 5.6E−01; p-value = 8.5E−03) is also known to be involved in the regulation of cerebellar development. During the first two postnatal weeks, *Ptn* is known to be associated with the control of granule cell migration. In addition, in adult mice, it controls neuronal plasticity by taking part in perineuronal nets [50].

We showed that H3K9 di-methylation is selectively increased in the cerebellar GCE layer in *Kdm3b*^+/−^ mice. Considering that the granule cells are major excitatory presynaptic inputs to Purkinje cells, Kdm3b may be critical for regulating the expression of genes involved in presynaptic function. Consistently, we found that the expression of *Cplx2* (log2 fold change = 5.9E−01; p-value = 1.70E−05), which is known to be related with synaptic transmission and plasticity, is significantly increased in *Kdm3b*^+/−^ mice [36, 37, 51, 52]. In the cerebellum, *Cplx2* is expected to be expressed at the terminal of granule cells (GCs) because it is considered as a maker for the excitatory presynaptic terminal in the brain [53]. Based on these, we speculate that an altered synaptic transmission or plasticity at GC-Purkinje cell (PC) synapse may result in deficits in the consolidation of OKR memory in *Kdm3b*^+/−^ mice. In addition, we previously reported that *Cplx2* expression is altered by oculomotor learning [20]. Hence, it would be interesting to elucidate the role of *Cplx2* in synaptic transmission at GC-PC synapses and in the consolidation of OKR memory. By providing a comprehensive list of molecules related to the trans-synaptic signaling pathway, we offer candidates for future studies on the synaptic control of cerebellum-dependent motor memory consolidation.

To our knowledge, this is the first study to present behavioral and molecular evidence implicating the critical role of histone modification in cerebellar learning. Interestingly, diverse epigenetic mechanisms have been implicated in several diseases affecting the cerebellum, including ataxia, autism spectrum disorders, Fragile X syndrome, and medulloblastoma [54]. Recent genetic studies have revealed *Kdm3b* as a risk gene for cognitive disorders, such as schizophrenia and intellectual disabilities [14]. Schizophrenia is highly associated with motor deficits, and cerebellar abnormalities are often reported in patients with schizophrenia [55]. Our study contributes to the understanding of the physiological and pathophysiological functions of the epigenetic regulator *Kdm3b* in the cerebellum.

## Supplementary Information

**Additional file 1:** Supplementary Figures.

**Additional file 2: **Supplementary Table 1. List of differentially expressed (DEX) genes (509 genes). 

**Additional file 3:** Supplementary Table 2. Gene ontology (GO) analysis of DEX genes. 

## Data Availability

All data generated or analyzed in this study are included in this published article. RNA-seq data are available from the Gene Expression Omnibus (https://www.ncbi.nlm.nih.gov/geo).

## Abbreviations

DEX: Differentially expressed
GO: Gene ontology
GC: Granule cell
GCE: Granule-cell-enriched
H3K9: Lysine 9 on histone 3
H3K9me1/2: Mono-and di-methylated lysine 9 on histone 3
OKR: Optokinetic response
PC: Purkinje cell
PCE: Purkinje-cell-enriched
H3K9me3: Tri-methylation on Histone 3 Lysine 9
VOG: Video-oculography
VOR: Vestibulo-ocular reflex
WT: Wild-type
