# Supplementary material for: Kdm3b haploinsufficiency impairs the consolidation of cerebellum-dependent motor memory in mice
Source: Mol Brain. 2021 Jul 3;14:106. doi: 10.1186/s13041-021-00815-5 (PMC8254933; doi:10.1186/s13041-021-00815-5)
Supplement: Supplementary file 1 — Additional file 1: Supplementary Figures. [file 13041_2021_815_MOESM1_ESM.pdf]

**Supplementary Information**

# Kdm3b haploinsufficiency impairs the consolidation of cerebellum-dependent motor memory in mice

Yong Gyu Kim[1, 2,*], Myeong Seong Bak[1, 2,*], Ahbin Kim[1, 2,*], Yujin Kim[3, 4], Yun-Cheol Chae[5], Ye Lee Kim[2, 6], Yang-Sook Chun[1, 2, 6], Joon-Yong An[3,4], Sang-Beom Seo[5], Sang Jeong Kim[1, 2, 7,+,] Yong-Seok Lee[1, 2, 7,+]

Affiliations

[1]Department of Physiology, Seoul National University College of Medicine, Seoul, 03080, Korea

[2]Department of Biomedical Sciences, Seoul National University College of Medicine, Seoul, 03080, Korea

[3]Department of Biosystems and Biomedical Sciences, College of Health Sciences, Korea University, Seoul 02841, Korea

[4]Department of Integrated Biomedical and Life Sciences, College of Health Sciences, Korea University, Seoul, Korea

[5]Department of Life Science, College of Natural Science, Chung-Ang University, Seoul 06974, Korea

[6]Ischemic/hypoxic disease institute, Seoul National University College of Medicine, Seoul 03080, Korea

[7]Neuroscience Research Institute, Seoul National University College of Medicine, Seoul, 03080, Korea

[*]These authors contributed equally to this work

[+]Correspondences: Sang Jeong Kim (Sangjkim@snu.ac.kr) and Yong-Seok Lee (yongseok7@snu.ac.kr)

**List of contents**

Supplementary Figure 1. Sagittal sections of Nissl stained *Kdm3b*$^{+/+}$ (a) and *Kdm3b*$^{+/-}$ (b) mice brain.

Supplementary Figure 2. Quantification of H3K9me2 level in the cerebellar flocculus between *Kdm3b*$^{+/+}$ and *Kdm3b*$^{+/-}$ groups.

Supplementary Figure 3. Comparison of relative Kdm3b mRNA level in the cerebellar flocculus between control and OKR-trained groups.

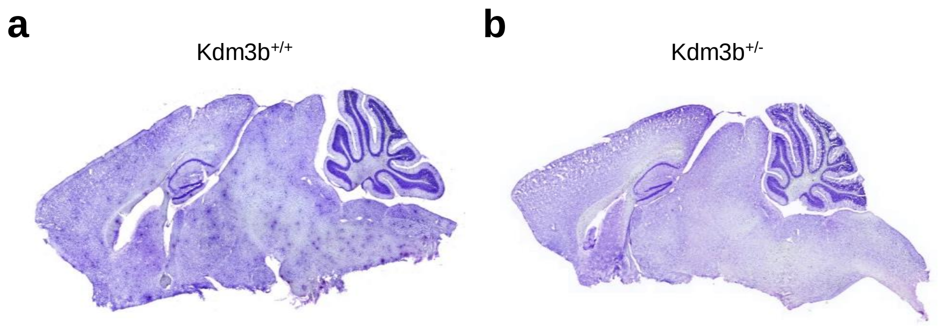


**Supplementary Figure 1**. Sagittal sections of Nissl-stained *Kdm3b*$^{+/+}$ (a) and *Kdm3b*$^{+/-}$ (b) mice brain.

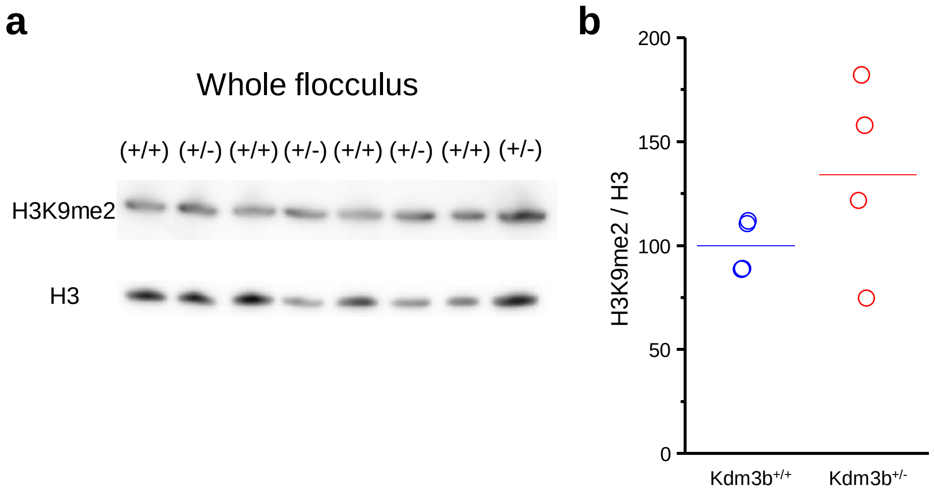


**Supplementary Figure 2.** Quantification of H3K9me2 levels in the cerebellar flocculus between *Kdm3b*$^{+/+}$ and *Kdm3b*$^{+/-}$ groups. (a) Immunoblotting bands of H3K9me2 and H3 in histone extracts from *Kdm3b*$^{+/+}$ and *Kdm3b*$^{+/-}$ mice (n=4 each group). (b) Quantification of immunoblotting in *Kdm3b*$^{+/+}$ and *Kdm3b*$^{+/-}$ groups.

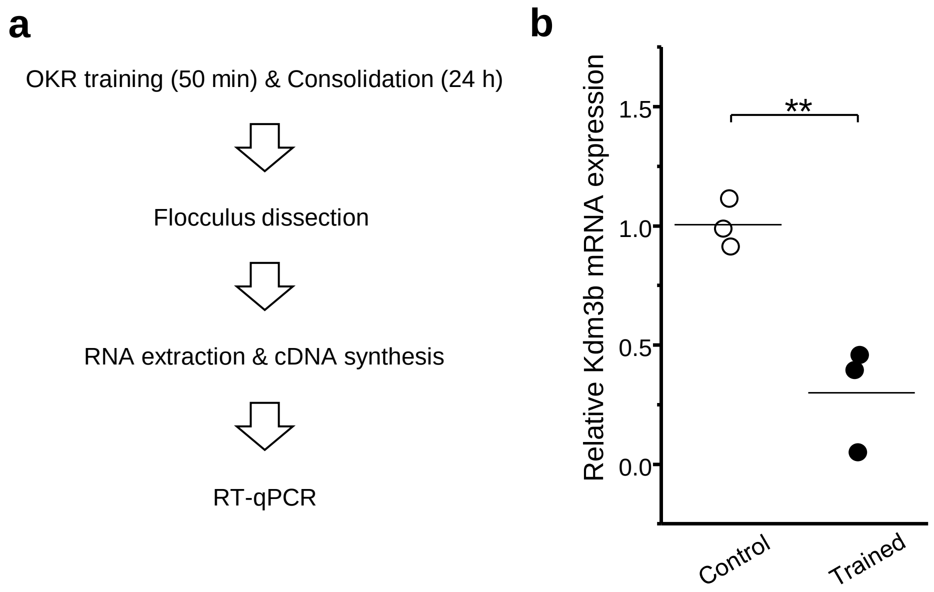


**Supplementary Figure 3.** Comparison of relative Kdm3b mRNA levels in the cerebellar flocculus between control and OKR training groups. (a) Experimental procedure. The flocculus was dissected after 50 min of OKR training and 24 h of consolidation in the dark. The extracted total RNA was reverse transcribed into cDNA. (b) RT-qPCR analysis was performed to compare Kdm3b mRNA expression levels between control and trained groups (n=3 per group). An independent t-test was performed to test statistical significance between the two groups. **$p < 0.01$.
